# Beyond Body Mass Index: Accurate Metabolic Disease‐Risk Phenotyping With 3D Smartphone Application

**DOI:** 10.1002/osp4.70025

**Published:** 2024-11-29

**Authors:** Cassidy McCarthy, Grant M. Tinsley, Sophia Ramirez, Steven B. Heymsfield

**Affiliations:** ^1^ Pennington Biomedical Research Center Louisiana State University System Baton Rouge Louisiana USA; ^2^ Department of Kinesiology and Sport Management Texas Tech University Lubbock Texas USA

**Keywords:** anthropometry, body composition, nutritional assessment, obesity

## Abstract

**Objective:**

Smartphone applications (apps) with optical imaging capabilities are transforming the field of physical anthropometry; digital measurements of body size and shape in clinical settings are increasingly feasible. Currently available apps are usually designed around the capture of two‐dimensional images that are then transformed with app software to three‐dimensional (3D) avatars that can be used for digital anthropometry. The aim of the current study was to compare waist circumference (WC), hip circumference (HC), four other circumferences (right/left upper arm, thigh) and WC/HC evaluated with a novel high‐precision 3D smartphone app to ground‐truth measurements made with a flexible tape by a trained anthropometrist.

**Methods:**

Forty‐four participants aged 20–78 years and body mass index 18.5–48.5 kg/m^2^ completed digital and manual circumference evaluations and dual‐energy X‐ray absorptiometry for visceral adipose tissue mass (VAT).

**Results:**

3D‐digital and ground‐truth tape WC, HC, and WC/HC estimates were highly correlated (*R* ^2^s, 0.90–0.97, *p* < 0.001), mean 3D and tape group means at each site did not differ significantly, mean absolute (± SD) and root‐mean square errors were low (e.g., WC, 3.4 ± 2.6 and 4.4 cm), and strong concordance correlations were present (0.90–0.99); bias with Bland–Altman analyses was small but significant (*p* < 0.001) for WC and WC/HC. Comparable results were observed for the four other circumferences. VAT was equally well‐correlated with 3D and tape WC measurements (*R* ^2^s 0.70, 0.69, both *p* < 0.001); comparable tape‐3D VAT‐WC/HC associations were also observed in males (*R* ^2^s, 0.85, 0.73, both *p* < 0.001) and females (*R* ^2^s, 0.43, *p* < 0.01; 0.73, *p* < 0.001).

**Conclusions:**

Digital anthropometry, with accessible technology such as the evaluated novel 3D app, has reached a sufficiently developed stage to go beyond body mass index for phenotyping patient's metabolic disease risks.

## Graphical abstract

This study reports the high accuracy of a novel three‐dimensional optical imaging smartphone application that provides a new opportunity to extend beyond BMI in evaluating and monitoring weight loss treatments in people with obesity.

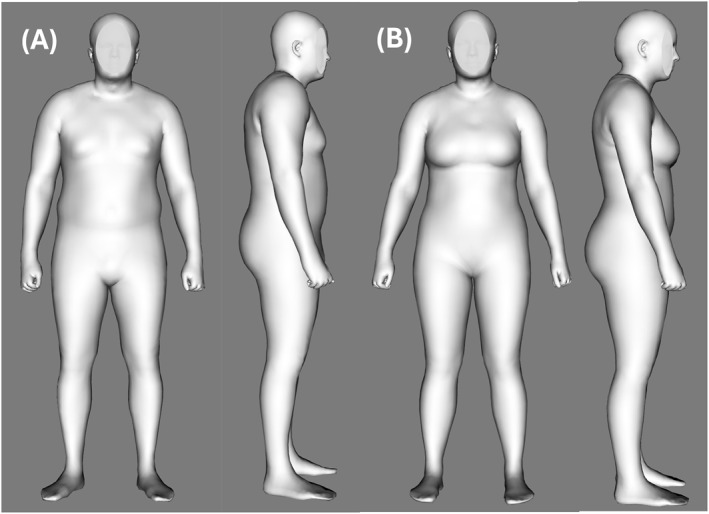

**Abbreviations**
BMI: body mass index
CCC: concordance correlation, coefficient
CV: coefficient of variation
DXA: dual‐energy X‐ray absorptiometry
MAE: mean absolute error
PC: principal component
RMSE: root mean square error

## 1 Introduction

Body mass index (BMI) is used by practitioners worldwide to phenotype weight status, although recent recommendations from major health organizations suggest acquiring additional measures that improve disease‐risk predictions [1]. Among these are the easy to quantify waist and hip circumferences (WC, HC) and the WC/HC ratio that are strongly linked to multiple clinical outcomes [2, 3, 4]. Despite supportive publications, including major organization guidelines, these anthropometric measurements are rarely quantified in clinical settings. Education of health care workers and technical training [5, 6, 7] improve the use and accuracy of flexible tape circumference measurements, but adoption in primary care and specialty medical facilities continues to be limited. Recent advances in smartphone technology and applications offer a new practical opportunity to go beyond BMI in clinical settings with the acquisition of digital anthropometric body dimensions [8]. Body size and shape estimates began with photographs obtained with digital cameras [9] and soon moved to two‐dimensional (2D) body images captured with smartphone cameras [10]. The 2D images could be converted to three‐dimensional (3D) avatars using neural network algorithms [10]. The derived 3D avatars could then be analyzed for key surface dimensions including WC and HC. A recent advance is the capability of obtaining a full 3D whole‐body image with a smartphone application [11]. The humanoid avatar obtained using this approach can be used to estimate waist and hip circumferences and the WC/HC ratio. Our experience with 2D smartphone applications indicated improved visual image resolution and precision with full 3D optical scans [11, 12]. The potential of this novel 3D digital anthropometric method led to the current study that aimed to establish the accuracy of evaluated body circumferences, including WC, HC, and WC/HC, relative to ground‐truth measurements acquired with a calibrated flexible tape by a skilled technician. Waist circumference and WC/HC are well‐recognized surrogate markers of visceral adipose tissue (VAT), a body compartment linked with metabolic disturbances associated with excess adiposity [2, 3, 4]. Accordingly, the current study was also designed to compare the strength of associations between VAT and WC and WC/HC to those manually measured with a flexible tape and the 3D smartphone application.

## 2 Methods

### 2.1 Study Design

A prospective sample of healthy adults (age ≥ 18 years) varying in sex, age, and BMI was recruited for this study. Each participant completed a medical examination, measurement of body weight and height, evaluation of six circumferences with a flexible tape and smartphone 3D scanner application (Prism Labs, Los Angeles, CA, USA), and estimation of percent fat and VAT with dual‐energy X‐ray absorptiometry (DXA; Discovery; Software version, Apex, 4.0.2. Hologic, Marlborough, Massachusetts).

The manually measured circumferences were set as ground‐truth for comparison to the corresponding six 3D digital circumference measurements (WC, HC; right and left thigh and mid‐upper arm). The associations between VAT evaluated with DXA and WC and WC/HC measured with the flexible tape and 3D optical application were explored.

### 2.2 Measurements

#### 2.2.1 Manual Circumferences

Body circumferences were measured (±0.1 cm) in triplicate by a single trained anthropometrist at the anatomic locations specified by the U.S. National Health and Nutrition Examination Survey (Table 1). A tension‐controlled tape was used for all circumference measurements (Gulick II, Lafayette Instrument Company, Lafayette, IN). The triplicate circumference measurements were averaged where no two measurements at the same location differed by more than 0.5 cm.

**TABLE 1:** Circumference measurement sites [22].

Circumference	Measurement site
Waist	Measured at the lateral border of the right ilium of the pelvis.
Hip	Measured at the maximum circumference point within the trochanteric area.
Upper arm (right and left)	Measured at the midpoint between the acromion process and tip of the elbow. This point was landmarked standing behind the subject as he or she held their arms at a 90° angle with palms facing up and then measured with arms relaxed at his or her sides.
Thigh (right and left)	Measured at the midpoint between the inguinal crease and the proximal boarder of the patella. This point was landmarked with the subject in a seated position with the legs positioned at a 90° angle and then measured in a standing position with a slight bend at the knee.

#### 2.2.2 Digital Circumferences

Participants, clothed in solid‐colored spandex or other comfortable tight‐form fitting clothing, swim cap, and women in sports bras were asked to stand erect facing the tripod‐mounted smartphone (iPhone 14 Pro, Apple Inc, Cupertino, CA) positioned 1.7 m away for a single head‐to‐toe scan. Participants' arms were positioned in an A‐pose and the mobile application provided real‐time guidance to ensure proper alignment by prompting forward or backward movement as needed. When activated, the 3D scanning application then requested the participant to self‐rotate 360° in place while 150 serial images were captured over 10 s with the device's built‐in front‐facing camera. Upon completion of the scan, participants were presented with a congratulatory screen on the application, indicating that the scan was successfully executed. Each scan was analyzed using machine learning for data pre‐processing through binary segmentation and obtaining frame‐to‐frame correspondences [11, 12]. Three‐dimensional humanoid avatars were produced through non‐rigid reconstruction. A parameterized body model was fitted to each avatar to normalize the pose to a canonical pose and to assure consistent measurement locations. The technical errors and intraclass correlation coefficients for repeated 3D scans were for WC 0.8%/0.995 and for HC 0.5%/0.995 in an earlier study [11]. The six circumferences were obtained from the generated avatar and reported through the scanning application.

#### 2.2.3 Dual‐Energy X‐Ray Absorptiometry

The DXA system was calibrated at regular intervals according to manufacturer specifications and participants were positioned on the scanning table according to standardized protocols. Two components were evaluated: fat mass (expressed in percentage of body weight) and VAT. Each participant had a single DXA scan with the National Health and Examination Survey mode activated. One female participant was missing DXA %fat data.

### 2.3 Statistical Methods

The ground‐truth flexible tape and 3D digital circumference measurements were compared with group means (± SD), mean absolute errors (MAEs, *X* ± SE), root‐mean square errors (RMSEs), concordance correlation coefficients (CCCs) [13], linear regression analysis (*R* ^2^), and Bland‐Altman analyses [13]. The associations between DXA‐measured VAT and WC and WC/HC acquired with the flexible tape and 3D application were evaluated using linear regression analysis. Analyses of the VAT‐WC/HC associations were conducted separately for males and females as there is a sexual dimorphism in these relationships [14]. Statistical analyses were conducted with Prism 10 (GraphPad, Boston, MA).

### 2.4 Institutional Review Board Approval

The study was approved by the Pennington Biomedical Research Center Institutional Review Board (IRB# PBRC 2022–002).

## 3 Results

### 3.1 Sample Characteristics

**FIGURE 1:**
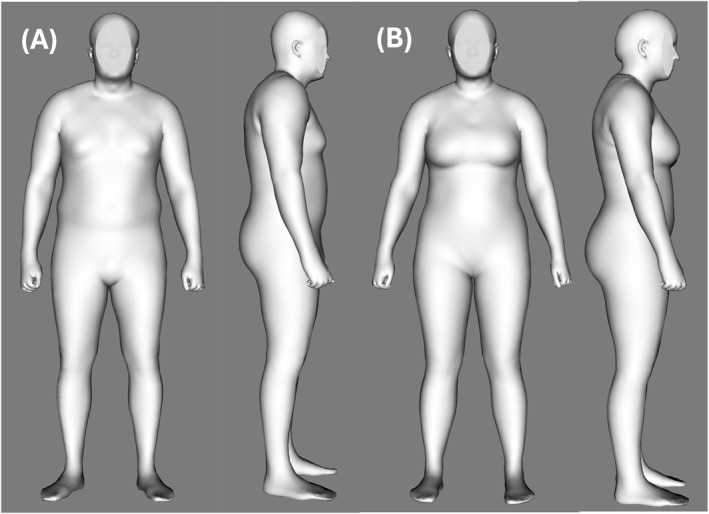
Representative acquired images of a male in Panel A and a female in Panel B.

The sample included 30 males and 14 females, mean age ∼50 years (range, 20–78 years) and BMI of ∼30 kg/m^2^ (range, 18.5–48.5 kg/m^2^) (Table 2). The males had a lower mean %fat (29.6% ± 7.9%) than the females (41.9% ± 8.4%). Representative digital 3D images of a male and female acquired with the application are shown in Panels A and B of Figure 1, respectively.

**TABLE 2:** Participant characteristics.

	Males	Females
*N*	30	14
Age (y)	48.4 ± 14.2	49.9 ± 14.2
Height (cm)	179.7 ± 6.9	163.6 ± 7.1
Weight (kg)	97.7 ± 24.5	80.3 ± 17.4
BMI (kg/m^2^)	30.3 ± 7.2	30.2 ± 7.7
Fat (%)	29.6 ± 7.9	41.9 ± 8.4^a^

*Note:* Results are *X* ± SD. Self‐identified race/ethnicity: White, *n* = 38; Black, *n* = 6.

Abbreviation: BMI, body mass index.

^a^ One female participant was missing %fat data and thus *n* = 13.

### 3.2 Circumference Evaluations

The results of the circumference comparisons between the 3D optical application and flexible tape are summarized in Table 3. There were no significant mean 3D optical‐tape differences between any of the circumferences, linear correlations were all strong (*R* ^2^s, 0.90–0.99; all *p* < 0.001), MAEs and RMSEs were low (1.5–3.4 cm; 1.9–4 cm), and the concordance correlation coefficients were all within a high range (0.90–0.99). All Bland‐Altman analyses were significant (slopes, *p* < 0.05–0.001) with small mean bias (−0.69–1.61 cm) except for HC and left arm circumference, for which bias was non‐significant.

**FIGURE 2:**
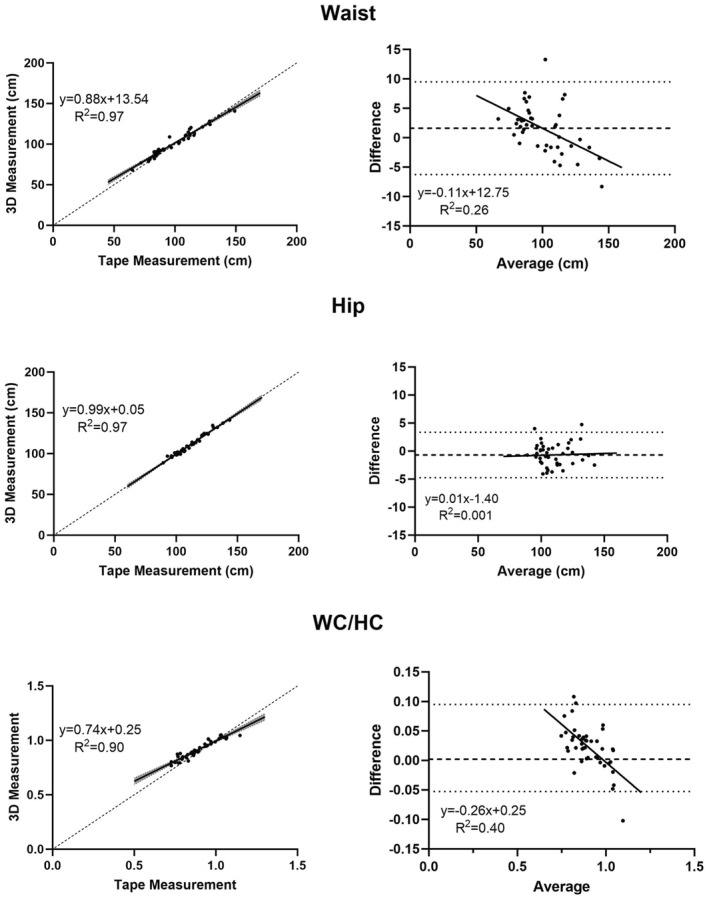
Waist and hip circumferences and the WC/HC ratio measured with the 3D optical smartphone application versus corresponding estimates made with a flexible tape by a trained anthropometrist; all correlations are *p* < 0.001 (left). Bland–Altman plots are shown to the right of each panel; horizontal lines are mean differences ± 95% confidence intervals (slopes of regression lines are *p* < 0.001 for WC and WC/HC). Additional details of these associations are presented in Table 1.

The associations between 3D optical and tape measurements are shown in Figure 2 for WC, HC, and WC/HC. As demonstrated in the figure and in Table 2, strong agreement was present between 3D optical and flexible tape waist and hip circumferences with both *R* ^2^s 0.97, respective CCCs of 0.97 and 0.99, and mean bias of ∼1%. The 3D optical‐tape association for WC/HC was also strong (*R* ^2^, 0.90) with non‐significant mean differences and a high CCC of 0.90. Bland‐Altman analysis indicated significant bias (*p* < 0.001) with a mean bias of  < 0.5%.

**FIGURE 3:**
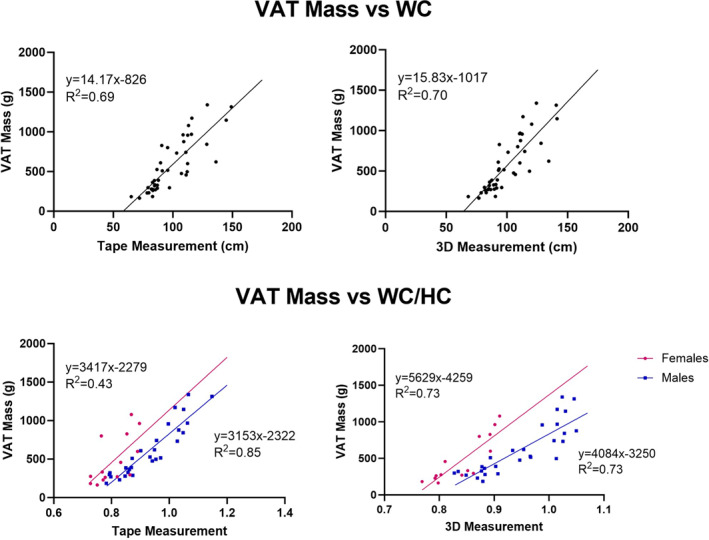
VAT mass as measured with DXA versus WC (upper panel) and WC/HC (lower panel) estimated manually with a flexible tape (left) and with the 3D smartphone application (right). One male with a technically inadequate DXA scan was excluded from this analysis. Both regressions in the upper panel are *p* < 0.001. VAT versus tape WC/HC in the lower panel, females, *p* < 0.01; other correlations are *p* < 0.001.

One male participant had a technically inadequate DXA scan for VAT and his results were excluded from the analyses. The correlations between VAT and WC, either by tape or 3D optical measurement, were similar with respective *R* ^2^s of 0.69 and 0.70 (both *p* < 0.001) (Figure 3). The correlations between VAT and WC/HC were also similar between manual and 3D optical measurements (males, *R* ^2^, 0.85 and 0.73; females, *R* ^2^, 0.43 and 0.73; VAT vs. tape WC/HC, females, *p* < 0.01; other correlations are *p* < 0.001).

**TABLE 3:** Results of digital circumference evaluations.

Circumference	Tape^a^ (*X* ± SD, cm)	3D optical (*X* ± SD, cm)	ΜΑΕ ± SD (cm)	RMSE (cm)	*R* ^2^ ^b^	CCC	Bias (*X* ± SD, cm)
Waist	99.2 ± 19.6	100.8 ± 17.5	3.4 ± 2.6	4.3	0.97	0.97	1.61 ± 4.02^d^
Hip	110.2 ± 12.7	109.5 ± 12.8	1.7 ± 1.3	2.2	0.97	0.99	−0.68 ± + 2.06
R thigh	58.7 ± 8.2	59.2 ± 6.7	2.0 ± 1.6	2.5	0.93	0.94	0.55 ± 2.50^d^
L thigh	58.1 ± 8.1	58.8 ± 6.4	1.9 ± 1.7	2.5	0.94	0.94	0.74 ± 2.45^d^
R arm	35.4 ± 5.3	34.9 ± 4.7	1.5 ± 1.1	1.9	0.89	0.93	−0.53 ± 1.82^c^
L arm	35.6 ± 5.2	34.9 ± 4.9	1.5 ± 1.2	1.9	0.88	0.93	−0.69 ± 1.80
WC/HC	0.90 ± 0.11	0.92 ± 0.08	0.03 ± 0.03	0.04	0.90	0.90	0.02 ± 0.04^d^

Abbreviations: CCC, concordance correlation coefficient; HC, hip circumference; L, left; MAE, mean absolute error; R, right; RMSE, root mean square error; WC, waist circumference.

^a^ Differences between flexible tape and 3D optical mean values do not differ significantly.

^b^ All correlations are *p* < 0.001.

^c^ *p* < 0.05.

^d^ *p* < 0.001 for Bland–Altman slopes.

## 4 Discussion

The current study evaluated the potential of a novel 3D smartphone optical scanning application to accurately provide six representative body circumferences relative to ground‐truth estimates acquired with a calibrated flexible tape by a trained anthropometrist. The findings of this investigation strongly support initial impressions from generated human avatars and precision studies [11, 12] that the evaluated 3D optical smartphone application provides surface measurements, in this case circumferences, that are overall equivalent to those acquired by a highly trained expert using a flexible tape similar to that advocated by many clinical guidelines [3, 4]. While there was bias relative to ground‐truth measurements for all 3D optical estimates except for HC and left arm circumference, the magnitude of these effects was small and has a plausible explanation: exact matching of digital and tape‐measured landmarks is extremely difficult. Even between‐observer differences in tape circumference measurements can be expected to show some degree of bias [6, 7, 15, 16]. One future option would be to adjust the 3D optical software to match human tape measurements, and an alternative would be to develop normative circumference values based on digital measurements. Whatever approach moves forward, the current study findings strongly suggest that digital anthropometry with advances such as those reported here opens new opportunities for clinical disease‐risk assessment.

The evaluated smartphone application had similar or better performance characteristics than a stationary 3D optical scanner (Size Stream, LLC; Cary, North Carolina, USA) currently in use at the investigators laboratory. Relative to reference tape measurements, the 3D research scanner had smaller *R* ^2^s (e.g., WC and HC, 0.91 and 0.95 vs. 0.97 and 0.97) and larger RMSEs (9.2 and 3.9 cm vs. 4.3 and 2.2 cm) in a sample comparable to the current study [17]. Improving smartphone camera technology has the potential to further improve the accuracy of digital anthropometric measurements [18].

While the current study focused on circumference measurements, their observed accuracy opens the possibility of estimating similarly accurate body lengths, volumes, and surface areas. These potential digital anthropometric dimensions can be incorporated into body composition prediction models and the developed 3D avatars can be used for patient educational purposes [19]. While the motivation for this study was to advance adiposity risk evaluations beyond BMI, changes in a person's shape and body composition can have diagnostic and monitoring value in pregnancy and disease states such as cancer and liver failure with ascites accumulation. Another opportunity is for patient home 3D optical evaluation and monitoring as is now often recommended for self‐measurement of WC [20].

An important current recognition is that associations with health risks are stronger for selected measures of body size and shape that go beyond BMI [2, 21]. These measures, such as the WC and WC/HC, are linked with high‐metabolic risk abdominal VAT. Findings from the current study showed that app‐derived WC and WC/HC were well‐correlated with those acquired by a highly trained anthropometrist and with VAT as measured with DXA. These observations extend earlier observations by Tinsley et al. showing good agreement (*R* ^2^, 0.81, *p* < 0.001; MAE, ∼3.5%) between the currently evaluated 3D application and percentage of body weight as fat [12]. Taken together, these observations support the potential of new digital applications, such as the one evaluated in the current report, as a means of phenotyping an adult's metabolic health risk.

While the results of this study for full 3D smartphone optical imaging are encouraging, data in this study were collected under ideal conditions in a well‐lit laboratory with participants clothed in tight‐fitting garments. Future implementation studies with larger samples are needed to evaluate mobile patient imaging applications in clinical and home settings. Patients may be sensitive to the acquisition of body images and application developers may therefore need to obscure avatar faces and linked personal information in collected scans housed on the web. While the current study participants had a wide range of ages and BMIs, future studies should ideally include large and equally diverse samples.

In sum, the current study indicated that full 3D images acquired with a smartphone application in adults varying widely in age and BMI yielded circumference values, notably WC, HC, and WC/HC, which are comparable to those quantified by an expert anthropometrist with a calibrated flexible tape. These observations, which extend earlier findings with 2D smartphone applications [8, 10], provide the impetus to move future studies into clinical and home settings.

## Author Contributions

C.M., G.M.T., S.R., and S.B.H. designed research, conducted research, provided essential materials, analyzed data, drafted the paper, and had primary responsibility for final content.

## Ethics Statement

The Pennington Biomedical Research Center Institutional Review Board approved the involved study and participants signed informed consent before commencing the evaluation protocol.

## Conflicts of Interest

S.B.H. reports on his role on the Medical Advisory Boards of Tanita Corporation, Amgen, and Medifast. G.M.T. has received research grants from Prism Labs, the manufacturer of the 3D scanning application evaluated in the present study, and in‐kind support through product loans or donations from Size Stream, Naked Labs, and Prism Labs.

## Data Availability

Data described in the manuscript will be made available upon request pending application and approval by the investigators.

## References

[1] S. Berg , AMA: Use of BMI Alone Is an Imperfect Clinical Measure, accessed May 23, 2024, https://www.ama‐assn.org/delivering‐care/public‐health/ama‐use‐bmi‐alone‐imperfect‐clinical‐measure#:~:text=The%20newly%20adopted%20AMA%20policy,applied%20on%20the%20individual%20level

[2] A. Criminisi , N. Sorek , and S. B. Heymsfield , “Normalized Sensitivity of Multi‐Dimensional Body Composition Biomarkers for Risk Change Prediction,” Scientific Reports 12, no. 1 (2022): 12375, 10.1038/s41598-022-16142-1. 35858946 PMC9300600

[3] S. Klein , D. B. Allison , S. B. Heymsfield , et al., “Waist Circumference and Cardiometabolic Risk: A Consensus Statement From Shaping America's Health: Association for Weight Management and Obesity Prevention; NAASO, the Obesity Society; the American Society for Nutrition; and the American Diabetes Association,” Diabetes Care 30, no. 6 (2007): 1647–1652, 10.2337/dc07-9921. 17360974

[4] R. Ross , I. J. Neeland , S. Yamashita , et al., “Waist Circumference as a Vital Sign in Clinical Practice: A Consensus Statement From the IAS and ICCR Working Group on Visceral Obesity,” Nature Reviews Endocrinology 16, no. 3 (2020): 177–189, 10.1038/s41574-019-0310-7. PMC7027970 32020062

[5] A. J. Dunkley , M. A. Stone , N. Patel , M. J. Davies , and K. Khunti , “Waist Circumference Measurement: Knowledge, Attitudes and Barriers in Patients and Practitioners in a Multi‐Ethnic Population,” Family Practice 26, no. 5 (2009): 365–371, 10.1093/fampra/cmp048. 19589884

[6] P. Sebo , S. Beer‐Borst , D. M. Haller , and P. A. Bovier , “Reliability of Doctors' Anthropometric Measurements to Detect Obesity,” Preventive Medicine 47, no. 4 (2008): 389–393, 10.1016/j.ypmed.2008.06.012. 18619998

[7] P. Sebo , D. Haller , A. Pechere‐Bertschi , P. Bovier , and F. Herrmann , “Accuracy of Doctors' Anthropometric Measurements in General Practice,” Swiss Medical Weekly 145 (2015): w14115, 10.4414/smw.2015.14115. 25701670

[8] S. B. Heymsfield , B. Bourgeois , B. K. Ng , M. J. Sommer , X. Li , and J. A. Shepherd , “Digital Anthropometry: A Critical Review,” European Journal of Clinical Nutrition 72, no. 5 (2018): 680–687, 10.1038/s41430-018-0145-7. 29748657 PMC6411053

[9] O. Affuso , L. Pradhan , C. Zhang , et al., “A Method for Measuring Human Body Composition Using Digital Images,” PLoS One 13, no. 11 (2018): e0206430, 10.1371/journal.pone.0206430. 30395607 PMC6218036

[10] M. D. Majmudar , S. Chandra , K. Yakkala , et al., “Smartphone Camera Based Assessment of Adiposity: A Validation Study,” NPJ Digital Medicine 5, no. 1 (2022): 79, 10.1038/s41746-022-00628-3. 35768575 PMC9243018

[11] G. M. Tinsley , C. Rodriguez , M. R. Siedler , et al., “Mobile Phone Applications for 3‐Dimensional Scanning and Digital Anthropometry: A Precision Comparison With Traditional Scanners,” European Journal of Clinical Nutrition 78, no. 6 (2024): 509–514, 10.1038/s41430-024-01424-w. 38454153

[12] G. M. Tinsley , P. S. Harty , M. R. Siedler , M. T. Stratton , and C. Rodriguez , “Improved Precision of 3‐Dimensional Optical Imaging for Anthropometric Measurement Using Non‐Rigid Avatar Reconstruction and Parameterized Body Model Fitting,” Clinical Nutrition Open Science 50 (2023): 40–45, 10.1016/j.nutos.2023.07.002.

[13] Lin L. I. K. , A. Hedayat , and W. Wu , Statistical Tools for Measuring Agreement (New York: Springer, 2012), 161.

[14] W. Shen , M. Punyanitya , A. M. Silva , et al., “Sexual Dimorphism of Adipose Tissue Distribution Across the Lifespan: A Cross‐Sectional Whole‐Body Magnetic Resonance Imaging Study,” Nutrition and Metabolism (London) 6, no. 1 (2009): 17, 10.1186/1743-7075-6-17. PMC2678136 19371437

[15] S. J. Ulijaszek and J. A. Lourie , “Intra‐ and Inter‐Observer Error in Anthropometric Measurement,” in Anthropometry: The Individual and the Population. Cambridge Studies in Biological and Evolutionary Anthropology, eds. S. J. Ulijaszek and C. G. N. Mascie‐Taylor (Cambridge: Cambridge University Press, 1994), 30–55.

[16] L. M. Verweij , C. B. Terwee , K. I. Proper , C. T. Hulshof , and W. van Mechelen , “Measurement Error of Waist Circumference: Gaps in Knowledge,” Public Health Nutrition 16, no. 2 (2013): 281–288, 10.1017/s1368980012002741. 22626254 PMC10271771

[17] B. Smith , C. McCarthy , M. E. Dechenaud , M. C. Wong , J. Shepherd , and S. B. Heymsfield , “Anthropometric Evaluation of a 3D Scanning Mobile Application,” Obesity 30, no. 6 (2022): 1181–1188, 10.1002/oby.23434. 35491718 PMC9177647

[18] C. H. Fenstermaker and Associates LLC , What Cell Phones Have Lidar?, accessed October 14, 2024, https://blog.fenstermaker.com/what‐cell‐phones‐have‐lidar/#:~:text=In%20the%20past%2C%20LiDAR%20systems,are%20gaining%20traction%20every%20day

[19] M. Horne , A. Hill , T. Murells , et al., “Using Avatars in Weight Management Settings: A Systematic Review,” Internet Interventions 19 (2020): 100295, 10.1016/j.invent.2019.100295. 31871900 PMC6909197

[20] L. B. G. Carranza , M. D. Jensen , J. J. Hartman , and T. B. Jensen , “Self‐Measured vs Professionally Measured Waist Circumference,” Annals of Family Medicine 14, no. 3 (2016): 262–266. 27184997 10.1370/afm.1896 PMC4868565

[21] B. M. Smith , A. Criminisi , N. Sorek , Y. Harari , N. Sood , and S. B. Heymsfield , “Modeling Health Risks Using Neural Network Ensembles,” PLoS One 19, no. 10 (2024): e0308922, 10.1371/journal.pone.0308922. 39383158 PMC11463747

[22] Centers for Disease Control and Prevention (CDC) . National Health and Nutrition Examination Survey (NHANES): Anthropometry Procedures Manual (National Center for Health Statistics (NCHS): Hyattsville, MD, 2007), https://wwwn.cdc.gov/nchs/data/nhanes/2007‐2008/manuals/manual_an.pdf.

